# Overview of Research Development on the Role of NF-κB Signaling in Mastitis

**DOI:** 10.3390/ani10091625

**Published:** 2020-09-10

**Authors:** Muhammad Zahoor Khan, Adnan Khan, Jianxin Xiao, Jiaying Ma, Yulin Ma, Tianyu Chen, Dafu Shao, Zhijun Cao

**Affiliations:** 1 State Key Laboratory of Animal Nutrition, Beijing Engineering Technology Research Center of Raw Milk Quality and Safety Control, College of Animal Science and Technology, China Agricultural University, Beijing 100193, China; zahoorkhattak91@163.com (M.Z.K.); dairyxiao@gmail.com (J.X.); majiaying@cau.edu.cn (J.M.); ma18810318038@163.com (Y.M.); 18355593440@163.com (T.C.); 2 Key Laboratory of Animal Genetics, Breeding, and Reproduction, Ministry of Agriculture & National Engineering Laboratory for Animal Breeding, College of Animal Science and Technology, China Agricultural University, Beijing 100193, China; dr.adnan93@cau.edu.cn; 3 Institute of Agricultural Information of CAAS, Beijing 100081, China; shaodafu@caas.cn

**Keywords:** mastitis, bovine mammary epithelial cells, inflammatory cytokines, NF-κB signaling, PRRs, TLRs

## Abstract

**Simple Summary:**

NF-κB signaling has been widely studied for its role in inflammatory and immunity-related diseases. Mastitis is considered one of the inflammatory and immunity associated diseases which are a serious threat to the global dairy industry. Having such a critical role in immunity and inflammation, NF-κB signaling is currently under target for therapeutic purposes in mastitis control research. The virulent factor, lipopolysaccharides (LPS), of bacteria after attachment with relevant Toll-like receptors (TLRs) on mammary epithelial cells starts its pathogenesis by using NF-κB signaling to cause mastitis. Several studies have proved that the blocking of NF-κB signaling could be a useful strategy for mastitis control.

**Abstract:**

Mastitis is the inflammation of the mammary gland. *Escherichia coli* and *Staphylococcus aureus* are the most common bacteria responsible for mastitis. When mammary epithelial cells are infected by microorganisms, this activates an inflammatory response. The bacterial infection is recognized by innate pattern recognition receptors (PRRs) in the mammary epithelial cells, with the help of Toll-like receptors (TLRs). Upon activation by lipopolysaccharides, a virulent agent of bacteria, the TLRs further trigger nuclear factor-κB (NF-κB) signaling to accelerate its pathogenesis. The NF-κB has an essential role in many biological processes, such as cell survival, immune response, inflammation and development. Therefore, the NF-κB signaling triggered by the TLRs then regulates the transcriptional expression of specific inflammatory mediators to initiate inflammation of the mammary epithelial cells. Thus, any aberrant regulation of NF-κB signaling may lead to many inflammatory diseases, including mastitis. Hence, the inhibiting of NF-κB signaling has potential therapeutic applications in mastitis control strategies. In this review, we highlighted the regulation and function of NF-κB signaling in mastitis. Furthermore, the role of NF-κB signaling for therapeutic purposes in mastitis control has been explored in the current review.

## 1. Introduction

Mastitis is the inflammation of the mammary gland, which is associated with pathological changes in udder tissue and decreases in the quantity and quality of milk [1,2]. Based on its duration and symptoms, mastitis might be acute or chronic [3,4]. Udder swelling, reduced milk yield, clots and increase somatic cell counts in milk are the most common clinical signs of mastitis [5]. All these factors are associated with pathogenic invasion, which is followed by the involvement of neutrophils under a specific stimulus. The inflammatory conditions may lead to chronic inflammation if not properly controlled and treated [6,7]. Different types of etiological invading bacterial pathogens are involved in bovine mastitis, of which *Coliforms, Escherichia coli*, *Streptococci* and *Staphylococcus aureus* are the most common bacteria [8,9,10,11]. Gram-negative bacteria, such as *E. coli*, can often cause clinical mastitis, and Gram-positive bacteria, such as *S. aureus*, are involved in subclinical mastitis infection [12,13,14].

Previous reports have documented that mammary epithelial cells work as the first line of defense of the mammary gland by generating multiple inflammatory cytokines against bacteria invading the epithelial cells [15,16]. Toll-like receptors (TLRs) are pattern recognition receptors (PRRs) expressed by many cell types, including mammary epithelial and immune cells [17]. In addition, it has been reported that innate immune systems recognize pathogens through TLRs [18,19,20].

The TLRs are distributed on the host cell surface that regulates the initial sensation of infection [21,22]. Every pathogen uses specific receptors on host cells—for example, *S. aureus* uses TLR2 and TLR6 [23], while *E. coli* utilizes TLR2 and TLR4—to transmit their signals inside the cell [21]. This specificity to TLRs depends on the virulent factor of pathogens. The cell wall of *S. aureus* is composed of lipoteichoic acid and peptidoglycan [24], while Gram-negative bacteria, such as *E. coli*, have lipopolysaccharides (LPS) in their cell wall [25]. The binding of pathogenic virulent factors to TLRs leads to the activation of several signaling components, including nuclear factor kappa-light-chain-enhancer of activated B (NF-κB) [26], which is considered one of the key players associated with inflammatory action. Besides, NF-κB signaling has been widely studied for its role in regulation of immunity and inflammation. Keeping in view the versatile functions of NF-κB signaling, the current review has specifically concentrated on summarizing possible research development on the role of NF-κB signaling activation and regulation of immunity and inflammation in bovine mastitis.

## 2. Materials and Methods

All studies which have discussed the role of NF-κB signaling in mammary gland infection, mainly bovine mastitis, were screened through authentic sources, such as PubMed, ScienceDirect, Web of Science, SpringerLink, Scopus and Google Scholar. The major keywords used for the search of literature were milk production, mastitis, NF-κB signaling, TLRs, MYD88, PPRs, cytokines, *E. coli-* and *S. aureus-*mastitis. The related data published in the English language in well-reputed peer-reviewed journals have been included for discussion in the current review. Furthermore, we excluded all content available in the form of conference abstracts, books, book chapters and unpublished findings.

## 3. General Regulatory Pattern of NF-κB Signaling

**Figure 1:**
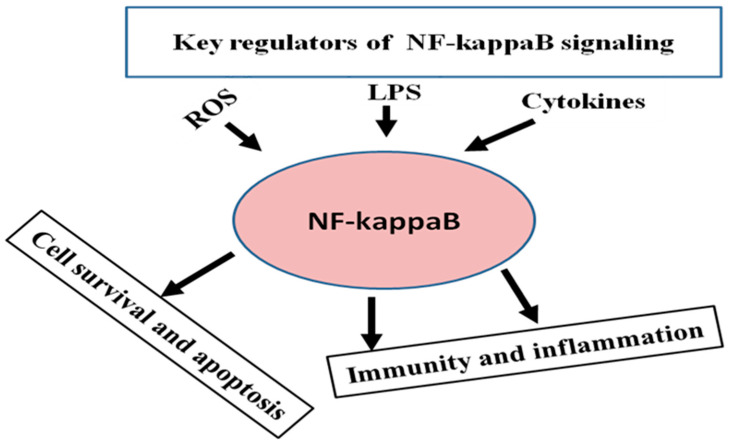
The key inducers of the NF-κB pathway and regulation of immunity, inflammation, cell survival and apoptosis by NF-κB signaling.

NF-κB is a common term used for inducible dimeric transcription factors. It is composed of a Rel family DNA binding protein which distinguishes common sequence motifs. Mammals express 5 Rel (NF-κB) proteins which are composed of two classes including Rel A (p65), c-Rel and Rel-B proteins which do not need proteolytic processing as the class is composed of NF-κB1 and NF-κB2 genes, encoded for p105 and p100, respectively, which do not require proteolytic processing to synthesize mature p50 and p52 NF-κB proteins [27]. The NF-κB protein was first found in murine B-lymphocytes, but currently, it has been identified in many cell types, including mammary epithelial cells [28]. Different external stimuli, such as tumor necrosis factor Alpha (TNF-α) [29], interleukin 1-beta (IL-1β) [30], LPS and reactive oxygen species (ROS) [31] after attachment with TLRs, activate NF-κB [32]. NF-κB signaling has an essential role in the regulation of immunity and inflammation [33], cell apoptosis, cell survival and proliferation (Figure 1) [34,35].

In addition, NF-κB signaling plays a vital role in the regulation of inflammatory cytokines, adhesion molecules, chemokines and growth factors involved in mammary gland inflammation [36]. Adhesion molecules are important proteins of tight junctions [37], which are closely related to the link between cell membranes and are required for normal lactation in mammals [38]. Song et al. has shown that LPS disrupt the permeability of the blood–milk barrier by activating the NF-κB signaling pathway. The pro-inflammatory cytokines regulated by the NF-κB signaling pathway promote the process of inflammation and interrupt the integrity of tight junction structures in the mammary epithelial cells [39]. The disruption in the blood–milk barrier has been reported during mastitis, which might be due to damage of the tight junctions responsible for normal lactation [40]. The disruption of tight junctions also may lead to loss of milk which is one of the common signs of mastitis in dairy cattle. Having such a critical role in inflammation and immunity, the NF-κB pathway has been widely targeted in mastitis research [41,42,43,44,45,46].

## 4. Role of NF-κB Signaling in Normal Physiology of Mammary Gland Development

A regulated pattern of activation of NF-κB during the various stages of the development of mammary glands has been demonstrated [46]. NF-κB activation rises during pregnancy and decreases during lactation, followed by elevation during the mammary gland involution, again [47,48]. This change in pattern suggests that NF-κB plays a significant role during pregnancy and involution. Mammary gland involution is associated with apoptosis of the secretory alveolar epithelium [49], and NF-κB has been explored to mediate the anti-apoptotic proteins [50]. These findings revealed the role of NF-κB in promoting the survival of epithelial cells [51]. It has been demonstrated that NF-κB activates the two essential lactogenic hormones, namely prolactin and oxytocin [52,53]. In addition to playing a role in the developmental process of normal mammary glands, NF-κB activation was found to be associated with mammary gland infections.

## 5. Role of NF-κB Signaling in Mastitis

The murine model and bovine reports have shown the link of NF-κB regulation with mastitis [43]. Most of the studies investigated the role of NF-κB in mastitis as a regulator of inflammatory cytokines [54,55]. Considerable losses of milk have been observed during mastitis and mammary gland involution which showed the link of both with the up-regulation of NF-κB during a time of milk loss and mammary gland remodeling.

### 5.1. Mechanism of NF-κB Signaling Activation by Bacteria during Mastitis

**Figure 2:**
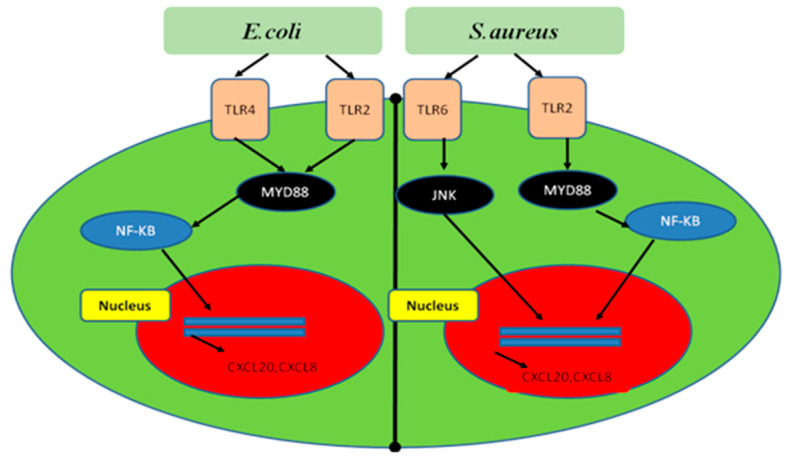
The interactive mechanism of *E. coli* and *S. aureus* with TLR2, TLR4 and TLR6, and the regulation of NF-κB signaling to activate the inflammatory genes.

LPS, a bacterial virulence factor, interacts with TLRs which are residing on surface mammary epithelial cells [56]. Upon activation, the TLRs further engage myeloid differentiation factor 88 (MyD88) [57] and c-Jun N-terminal kinase (JNK) [58], which triggers NF-κB [32] and mitogen-activated protein kinase (MAPK) signaling. The translocation of NF-κB and MAPK signaling further regulates the production of target inflammatory genes [59,60,61,62]. The mechanism of NF-κB signaling activated by *S. aureus* and *E. coli* during mastitis is shown in Figure 2.

### 5.2. Mechanism of NF-κB Signaling Activation by Inflammatory Cytokines

**Figure 3:**
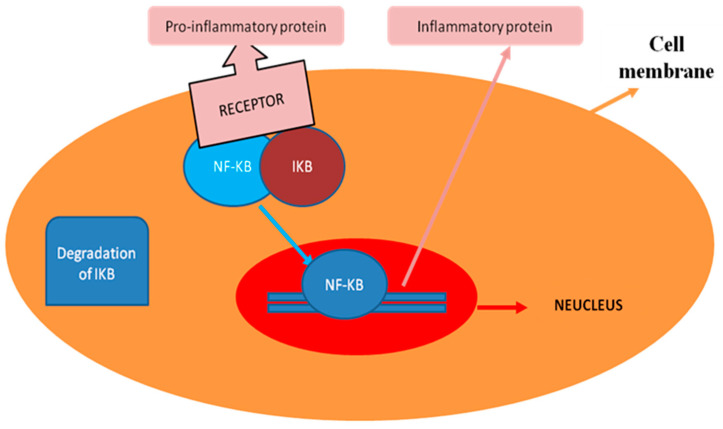
The regulation of the inflammation process by pro-inflammatory cytokines through NF-κB signaling; the cytokines, after attachment with receptors, cause the degradation of IKB from NF-κB. Upon activation, NF-κB directly binds to the promoters of target genes on DNA in the nucleus and regulates the specific inflammatory proteins.

Besides *S. aureus* and *E. coli,* various inflammatory cytokines activate NF-κB signaling regulation in mammary epithelial cells. The NF-κB and MAPK pathways activate pro-inflammatory cytokines interleukin 6 (IL-6), IL-1β and TNF-α [63]. Nuclear factor-κB is a nuclear transcription factor that exists in an inactive form in the cytoplasm and is bound to its inhibitor IκB [64,65]. Once activated, the NF-κB unit p65 separates from IκB and translocates from the cytoplasm to the nucleus, where it regulates inflammatory gene expression [66]. The pathogenic message usually causes the liberation of NF-κB from IκB [65]. The regulation of the inflammation through NF-κB by pro-inflammatory cytokines is shown in Figure 3. The promoter of the inflammatory genes contains binding sites for NF-κB, and thus mostly depends on NF-κB for its regulation [67]. It has been reported that active NF-κB complexes cannot be detected in healthy cow milk cells, while the NF-κB elevated level was noticed in the milk cells of cows with acute mastitis. In addition, the activity of NF-κB in milk cells varies from low to high in chronic mastitis [67]. Stimulation of LPS causes mammary epithelial cells to produce cytokines TNF-α, IL-6 and IL-1β [68]. The increased levels of TNF-α, IL-6 and IL-1β have been observed in LPS-infused mammary glands [69]. Furthermore, Blum et al. reported the high level of cytokines (TNF-α, IL-6 and IL-17), somatic cell count (SCC), and up-regulation of TLR4 expression in leukocytes of the milk of an *E. coli*-induced mastitic cow [70]. In the mammary glands, inflammation is associated with an increased level of neutrophil chemo-attractants and the cytokines IL-1ß, IL-6, IL8 and TNF-α [71,72]. The expression level reported for IL8 and TNF-α in *E. coli* induced-mastitis in bovine mammary epithelial cells (BMECs) was much higher than for *S. aureus,* which is due to the weak Lipoteichoic acid (LTA) induction of TNF-α, or inactivation of NF-κB signaling [73]. Boulanger et al. observed that NF-κB was highly associated with the level of the expression of interleukin-8 and granulocyte/macrophage colony-stimulating factors, two NF-κB-dependent cytokines critically linked to the regulation and continuation of neutrophilic inflammation. Altogether, these findings suggested the crucial role of NF-κB in the pathogenesis of mastitis.

### 5.3. Bovine Myeloid Differentiation Primary Response 88 (MYD88), NFKBIA and TRAPPC9 Role as a Regulator of Lipopolysaccharide (LPS)-Induced NF-κB Signaling Pathways

MYD88 is the main adopter molecule for TLR2, 4, 5, 7, 8 and 9 signaling [74]. The TLRs, when activated by mastitis-induced bacteria, pass the signal to MYD88, which is considered the critical immune regulator adapter molecule against various pathogens [75,76]. MYD88 acts as the key regulator of NF-κB by causing the degradation of IKB. Wang and his co-authors compared the expression level of MYD88 in healthy and mastitic cows. It was observed that MYD88 expression, which works as a bridge between TLRs and NF-κB, was elevated in mastitic cows compared to healthy ones [69,77,78]. It was noticed in a study that inhibition of MYD88, along with its inhibitor, Pepinh-MYD, significantly reduced the level of NF-κB [63].

Another essential protein is the nuclear factor of kappa light polypeptide gene enhancer in B-cells inhibitor, alpha (NFKBIA), which encodes IκB and is responsible for the negative activation of NF-κB transcription factors. It has been shown in a report that LPS cause the degradation of IκBα; they facilitate the translocation of NF-κB in the nucleus, which in response accelerates the re-synthesis of IκBα [79]. Fang et al. noticed the up-regulation of NFKBIA in *S. aureus*-induced mastitis [80]. The trafficking protein particle complex 9 (TRAPPC9), also called NIK-and-IKK2-binding protein (NIBP), is a key regulator of NF-κB signaling [72,81,82]. An in-vitro study revealed NIBP low expression results in the down-regulation of TNF-α-induced NF-κB [83]. Wang et al. noticed through a genome-wide association study (GWAS) that the mutation in TRAPPC9 is associated with milk SCS [84]. The high expression level of the TRAPPC9 gene was reported in mammary epithelial cells infected with *S. aureus*. Furthermore, it was revealed that the TRAPPC9 gene might be considered a potential marker against mastitis [85]. The above-published studies showed that MYD88, NFKBIA and TRAPPC9 might work as a bridge between cell surface receptors and NF-κB. Thus, any change in these genes may disturb NF-κB signaling, which facilitates the pathogenesis of mastitis.

### 5.4. NF-κB Regulates the Immunity and Inflammatory Linked Genes during Mastitis

When NF-κB signaling is activated by external stimuli, such as bacteria or cytokines, it starts to regulate the production of inflammatory chemokines (IL-8, CXCL1, CXCL10, etc.), cytokines (IL-6, TNF-α, IFN-gamma and IL-1β), adhesion molecules (ICAM-1 and MMPs), growth factors (CSF) and apoptotic associated genes [76,86,87]. For the site of infection, many proteins are required; these proteins are: adhesion factors, such as ICAM-1 and VCAM-1, which facilitate neutrophil margination, diapedesis and transepithelial migration; chemokines, such as interleukin (IL)-8, which are responsible for chemotactic of neutrophils; IL-1*β* and TNF-*α*, which regulate neutrophils [67]. When bacteria enter the teat, the mammary epithelial cells secrete chemokines (CXCL8 and CXCL20) and cytokines (TNF-α and IL-1β). Production of cytokines and chemokines in the milk of the mastitic mammary gland is considered the key player of inflammation [72,88]. The TNF-α and fatty acid synthetase (FAS) mRNA expression was significantly up-regulated in LPS-challenged quarters [89]. A study reported the up-regulation of CXCL8 and TNF-α in *E. coli* induced mastitis in mammary epithelial cells [88]. In addition, the high expression of CXCL10, CCL2, CCL5 *and* CCL20 was noticed in bovine mammary epithelial cells in *E. coli* induced mastitis, which is essential for the recruitment of leucocytes [90]. The expression levels of IL-6, complement factor 3 (C3), NFKBIA and MMP9 were also elevated during mammary gland infection [90]. It has been reported that monocytes, natural killer cells and activated lymphocytes are majorly regulated by the chemokines CXCL10 and CCL5 [91]. Apart from the above functions, CXCL10 directs the recruitment and activation of neutrophils towards LPS-infection spots in mice and humans [83,92,93]. In addition, CXCL10 was reported as a highly expressed gene in response to *E. coli* infection in mammary glands [94]. The levels of CXCL10 and ICAM1 were noticed to be significantly elevated in the *S. aureus*-mastitic mammary glands of cows [95]. Similarly, the high expression of CCl5 has also reported in *E. coli*-induced mastitis in BMECs [96]. The expression levels of CXCL8, IL6 and CSF3 were higher in *S. aureus* challenged BMECs [80]. Additionally, many other immunity and inflammatory associated genes, such as SAA3, CCL5, C3 and CSF3, were also documented in mastitis-infected mammary glands [69]. Furthermore, the high expressions of CXCL10, IL6, CXCL8, IFN-gamma and IL-1β induced by LPS in BMECs are able to regulate inflammation [97]. It has been demonstrated in previous reports that inflammatory cytokines and chemokines create protection against foreign invading pathogens in bovine mammary glands, by increasing the movement of leucocytes from the blood into the mammary tissue [98]. Similarly, a study reported the protecting role of IL-1β by recruiting neutrophils into the mammary gland [99].

### 5.5. Research Progress on Target of NF-κB Signaling as a Therapeutic in Mastitis Control

It is well known that TLRs, upon recognition of external stimuli, activate NF-κB regulation to produce inflammatory linked genes to eliminate the cause of infection in mammary epithelial cells. TLR4, a pro-inflammatory cytokine, and LPS, a component of the cell wall of bacteria, are common inducers of NF-κB signaling. The LPS-induced inflammation in mammary epithelial cells causes the up-regulation of TLR4 [100,101]. Recently, NF-κB signaling is being widely targeted as a therapeutic choice against mastitis resistance. A study proved, experimentally, that selenium restricts *S. aureus*-induced mastitis through inhibition of the MAPK and NF-κB pathways and TLR2 [102]. Cytokines, an important group of inflammatory mediators, play a major role in the process of inflammation [103]. Stimulation by LPS causes mammary epithelial cells to produce the cytokines TNF-α, IL-6 and IL-1β [60]. Increased levels of TNF-α, IL-6 and IL-1β have been observed in LPS-infused mammary glands. Similarly, Akhter et al. [104] noticed the up-regulation of pro-inflammatory cytokines in *S. aureus*-induced mammary epithelial cells. Further, they proved that the expression levels of genes associated with TLR2/TLR4-mediated NF-κB/MAPKs pathways were higher in *S. aureus*-infected mammary epithelial cells. The excessive expression of pro-inflammatory IL1β may lead to pathological conditions [105]. Dai et al. noticed that methionine and arginine attenuated the proinflammatory action by preventing the regulation of NF-κB. Furthermore, methionine and arginine down-regulated the levels of TLR4 and IL1β in LPS-induced mastitis, which caused the excessive regulation of inflammatory changes, and thus damaged the cells [106]. Taken together, it has been concluded here that methionine and arginine, being blockers of NF-κB, can be considered as prophylactic agents of mastitis.

Exogenous hydrogen sulfide has the ability to suppress inflammatory cytokine production, ROS [107,108,109], and promotes anti-inflammatory proteins [110]. The high level of ROS is associated with the imbalance between cellular redox states and oxidative stress, which has a significant role in the promotion of inflammation [111]. It was noticed that LPS alone diminished cell viability and caused inflammatory changes in mammary epithelial cells. However, it was found that the hydrogen sulfide (H2S) combined with LPS restored the viability of the cells [112]. Sun et al. revealed that H2S, after entry into the cells, first blocked the TLR4 and ROS, and thereby no signal was given for NF-κB to produce a high level of inflammatory proteins in mammary epithelial cells [113]. In addition, the mRNA expression of TNF-α, IL-1β, IL-8 and IL-6 was also very low in H2S-treated mammary epithelial cells.

Morin has anti-inflammatory properties [114] and inhibits the release of the inflammatory cytokines IL-6 and IL-8 and tumor necrosis factor (TNF) from mast cells [115]. It was experimentally proved that morin is associated with inhibition of TNF-α, IL-6 and IL-1β in LPS-induced bovine mammary epithelial cells (bMECs). To suppress the level of cytokines, morin down-regulates the levels of MAPK and NF-κB pathways in LPS-induced mammary epithelial cells [78]. NLRP3 inflammasome is the key regulator of IL-1β, while a recent study noticed that morin significantly down-regulated the level of IL-1β [116] in LPS-induced bovine mammary epithelial cells by suppression of NF-κB and nucleotide-binding domain, leucine-rich repeat-containing family, pyrin domain containing 3 (NLRP3) inflammasome [117]. Furthermore, it has been noticed that morin also maintained the integrity of the tight junction from the action of the inflammatory cytokines regulated by NF-κB [63]. Likewise, polydatin has anti-inflammatory efficiency and can be used to control *S. aureus*-induced mastitis. The most in-depth mechanism showed that polydatin decreased the expression of TLR2 and MyD88, which further suppressed the level of NF-κB in mammary epithelial cells of *S. aureus-* induced mastitis [60].

Tea tree oil (TTO) is an essential oil which has antibacterial and anti-inflammatory properties and promotes the movement of polymorphonuclear leukocytes towards the infection. TTO also inhibits NFKBIA and TNF-α [118]. In addition, TTO act as an inhibitor of the NF-κB pathway, which is essential for the regulation of immunity and inflammatory responses in mammary glands. Nucleotide-binding oligomerization domain (NOD) is a type of PRR that plays an important role in the regulation of innate immunity [119]. Recently, it was documented that by blocking NOD1/NF-κB signaling, LPS stimulation reduced neutrophil migration and phagocytic killing ability. Further, it was proved that the activation of NOD1/NF-κB in vitro restricted the action of LPS by promoting the functional capacity of neutrophil [60]. Chlorogenic acid has anti-inflammatory and antibacterial effects [120,121]. A study reported that chlorogenic acid inhibits cytokine production in LPS-stimulated RAW264.7 cells by suppressing the phosphorylation of NF-κB [122]. Similarly, chlorogenic acid was noticed to reduce the level of cytokines followed by inhibition of TLR4 and phosphorylation of NF-κB in LPS-induced mastitic mammary epithelial cells [123]. Thymol was found to be very effective in mastitis treatment. The mechanism for the association of thymol was tested in BMECs. The western blot result showed that thymol treatment significantly inhibited the production of IL-6 and TNF-α, followed by suppression of the NF-κB pathway [124]. In Table 1, we summarized those studies which targeted the NF-κB signaling to control mastitis.

From the above discussion, it has been cleared that NF-κB signaling plays a role of backbone in the pathogenesis of mastitis by promoting cytokine production. Thus, by targeting NF-κB, mastitis can be effectively controlled [124].

## 6. Conclusions

Overall, the current review, based on published studies, revealed that activation of NF-κB resulted in decreased of milk and apoptotic signaling, which could be minimized through selective modulation of NF-κB signals. Furthermore, the review suggested that NF-κB is a vital regulator of milk loss during mammary gland involution and infection, and recognized the NF-κB signaling pathway as a possible target for preventing mastitis-induced milk loss in dairy cattle. In addition, based on published literature, we concluded that TLR4, IL-1β, IL-6, TNF-α and MYD88 are key players in NF-κB signaling and also have an essential role in mastitis development. From the literature studies, it was revealed that *S. aureus* and *E. coli,* after attachment with TLRs, used NF-κB pathway for pathogenesis. Thus, the utilization of NF-κB as a therapeutic target in mastitis control showed successful outcomes. In addition, TLR4, IL-1β, IL-6, TNF-α, MYD88 and NF-κB might be a useful addition as markers in mastitis control strategies.

## Figures and Tables

**Table 1:** Chemicals and their anti-inflammatory effect in mastitis by suppression NF-κB signaling.

Authors	Agent	Function	Targets
Sun et al. [113]	H2S		Block TLR4, ROS, NF-κB
Garcia et al. [125]	Citrus oils	Antibacterial	Down-regulate TLR2, NFKBIA, IL8, TNF-α
Wang et al. [78]	Morin	Anti-inflammatory	Inhibit IL-6, TNF-α, IL-1β, suppress NF-κB phosphorylation
Li et al. [126]	8-Methoxypsoralen	Anti-inflammatory	Inhibit IL-6, TNF-α, IL-8, IL-1β, suppress NF-κB phosphorylation
Chen et al. [36]	Nuciferine	Anti-inflammatory	Inhibit TLR4, TNF-α, IL-1β, suppress NF-κB phosphorylation
Yang et al. [127]	Oxymatrine	Anti-inflammatory	Suppress NF-κB phosphorylation
Ershun et al. [128]	Cepharanthine	Anti-inflammatory	Inhibit IL6, TNF-α, IL-1β, suppress NF-κB phosphorylation
Su et al. [129]	Rutin		Decrease level of IL-1β, IL-6, and TNF-α, suppress NF-κB phosphorylation
Liu et al. [112]	Sodium houttuyfonate	Antinflammatory	Inhibit NF-κB phosphorylation
Li et al. [130]	Emodin ameliorates	Anti-inflammatory, antibacterial	Decrease level of IL-1β, IL-6, and TNF-α, suppress NF-κB phosphorylation
Hu et al. [42]	Cynatratoside-C from Cynanchum atratum	Anti-inflammatory	Suppress TLR4, inhibit NF-κB phosphorylation
He et al. [131]	Docosahexaenoic acid	Anti-inflammatory	Decrease level of IL-1β, IL-6, and TNF-α, suppress NF-κB phosphorylation
He et al. [132]	Baicalein	Anti-inflammatory	Suppress TLR4, inhibit NF-κB phosphorylation
